# Gaucher disease type 3c: Expanding the clinical spectrum of an ultra‐rare disease

**DOI:** 10.1002/jmd2.12440

**Published:** 2024-08-15

**Authors:** John S. Wang, Rebecca L. Koch, Daniel Kenney‐Jung, Erin Huggins, Sirajbir S. Sodhi, Andrew P. Landstrom, Dilraj S. Grewal, Priya S. Kishnani

**Affiliations:** ^1^ School of Medicine Duke University Medical Center Durham North Carolina USA; ^2^ Department of Pediatric Medical Genetics Duke University Durham North Carolina USA; ^3^ Children's Health Center Neurologic Clinic Duke University Medical Center Durham North Carolina USA; ^4^ Department of Pediatrics, Division of Cardiology and Department of Cell Biology Duke University School of Medicine Durham North Carolina USA; ^5^ Department of Ophthalmology Duke University Medical Center Durham North Carolina USA

**Keywords:** beta‐glucocerebrosidase, enzyme replacement therapy, Gaucher disease type 3c, *GBA*, neuropathy, uveitis

## Abstract

Gaucher disease (GD) type 3 is an autosomal recessive lysosomal disease caused by deficiency of β‐glucocerebrosidase (GCase) and encompasses a spectrum of cardiac, neurological, and ophthalmological abnormalities. Although the clinical presentations can be diverse, a recognized clinical trajectory points to an early onset, predominantly before 18 years. GD type 3c is primarily caused by homozygosity for *GBA* pathogenic variant c.1342G>C (p.Asp448His; historically referred to as D409H) and includes visceral, hematological, skeletal, and cardiac abnormalities. Notably, GD type 3c is distinct from other GD types because it is primarily characterized by valvular heart disease. Yet, with less than 50 patients with GD type 3c reported to date, the phenotypic spectrum and extent of cardiac involvement remains ill‐defined. We present a 20‐year‐old female with an atypical presentation of GD type 3c consisting of chronic intermediate uveitis as the presenting feature and the presence of extensive polyneuropathy starting in adolescence which has been previously unreported in GD type 3c. Distinctively, she has maintained normal cardiac function. Moreover, we compare our case with those reported in the literature to broaden awareness of the varied initial presentations of this disease. The diverse presentations seen in GD type 3c, underscored by our case and those previously reported, demonstrate the need for standardized evaluation and management protocols.

**Synopsis**

Despite Gaucher disease type 3c being primarily characterized by valvular heart disease, we present an adult female patient that presented with chronic intermediate uveitis and polyneuropathy and has maintained normal cardiac function.

## 1 INTRODUCTION

Gaucher disease (GD) is an autosomal recessive lysosomal disease (LD) caused by a deficiency of β‐glucocerebrosidase (GCase) due to biallelic variants in the *GBA* gene. GCase deficiency results in accumulation of glucocerebroside in the lysosomes of macrophages. These affected macrophages (“Gaucher cells”) infiltrate the bone marrow, spleen, liver, and in some instances the brain causing bone lesions, cytopenia, splenomegaly, hepatomegaly, and neurological damage. GD is classified into 3 subtypes. GD type 1 (OMIM 230800) is the most prevalent in Europe and North America (1 in 40 000 live births) and is associated with normal or near‐normal life expectancies. Compared with GD type 1, GD types 2 and 3 have a spectrum of neurological involvement.^1^ Individuals with GD type 2 (OMIM: 230900) exhibit rapid, progressive brainstem degeneration and typically die by 2 years of age. Affected patients often present prenatally with hydrops fetalis and/or motor regression and cranial nerve dysfunction within the first 6 months of life.^2^, ^3^ GD type 3 (OMIM 231000) have variable neurological involvement and disease progression is typically slower with longer survival^4^ compared with patient with GSD type 2, overall representing a disease continuum.

GD type 3 is divided into several subtypes: 3a, 3b, and 3c.^5^ There is also a Norrbottnian form of GD type 3 common in individuals of northern Swedish ancestry.^6^ All GD type 3 subtypes typically exhibit neurological involvement, most often consisting of ocular motility disorder presenting as horizontal supranuclear gaze palsy and slowed saccades.^5^ In addition, patients with GD type 3a may display cognitive impairment, auditory processing defects, seizures, muscle weakness, ataxia, and, in some instances, a progressive myoclonic epilepsy. GD type 3b is associated with systemic involvement characterized by enlarged liver and spleen, anemia, thrombocytopenia, bone manifestations such as kyphosis, infiltrative lung disease, and horizontal supranuclear gaze palsy as the major neurological complications.^7^ GD type 3c is distinct from GD types 3a and 3b, with its own separate entry in OMIM (231005). It is primarily characterized by valvular heart disease either with or without progressive calcifications which is not observed in other forms of GD.^8^ There are some patients with GD type 3 with no evidence of neurological involvement.^9^

To date, less than 50 patients with GD type 3c have been described in the literature worldwide. It is primarily caused by homozygosity for NM_000157.4 (*GBA*) pathogenic variant c.1342G>C (p.Asp448His; historically referred to as D409H). Neurological manifestations include hydrocephalus and/or ventriculomegaly (45%), brain imaging abnormalities (32.5%), cognitive and psychological involvement (17.5%), spasticity and hyperreflexia (12.5%), seizures (10%), and sensorineural hearing loss (10%).^9^ A unique finding in patients with GD type 3c is aortic and mitral valve disease with one report detailing a median age of onset at around 15.34 years.^9^, ^10^ Approximately 77.5% of patients experience calcifications of the valves and 15% have other artery involvement.^10^ These complications can result in myocardial ischemia, cardiomyopathy, and heart failure. There are also rare reports of arrhythmias and conduction delay^11^ that lead to cardiac arrest and potentially death, which may represent a more severe phenotype of cardiac involvement. Yet, the full phenotypic spectrum and extent of cardiac involvement of GD type 3c remains ill‐defined due to a paucity of reported patients in the literature.

We present a 20‐year‐old patient homozygous for c.1342G>C with an atypical presentation of GD type 3c consisting of chronic intermediate uveitis as the presenting feature, presence of extensive polyneuropathy starting in adolescence, and the absence of overt cardiac involvement. We then compare our case to all reported GD types 3c cases to broaden clinicians' awareness of varied initial presentations of this disease. The detailed account of our patient's distinctive phenotype significantly enhances the understanding of phenotypic diversity in GD type 3c.

## 2 CASE REPORT

**FIGURE 1:**
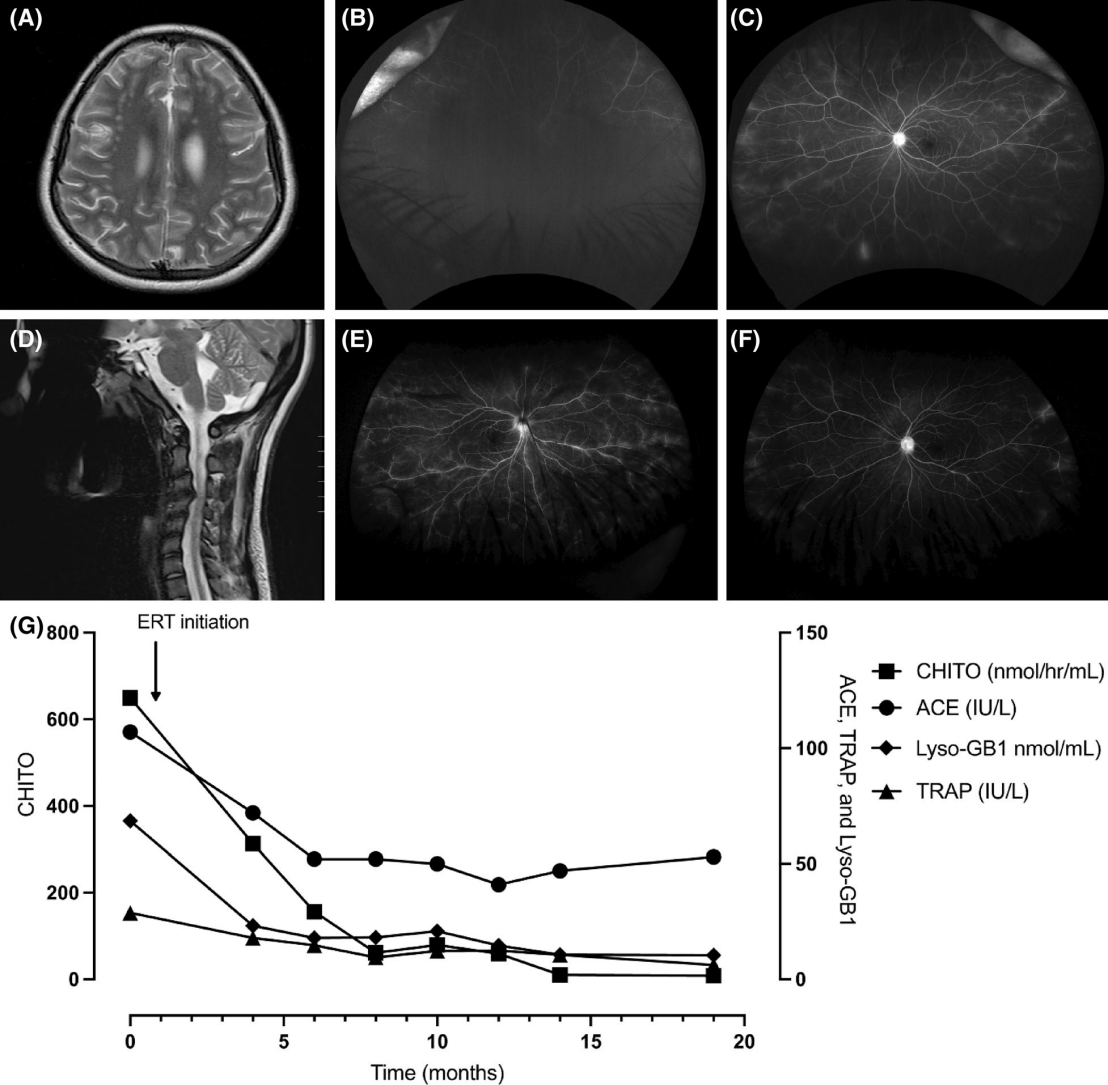
Magnetic resonance imaging (MRI) of brain and spinal cord, fluorescein angiograms, and biomarker evaluations of our patient with Gaucher disease type 3c before and after enzyme replacement therapy initiation. At age 14 years, (A) the brain MRI, T2 revealed multiple nonspecific small foci of white matter T2/FLAIR hyperintensity within right greater than left cerebral hemispheres. Additionally, (B) the right eye details were obscured due to vitreous hemorrhage and (C) the left eye showed mid peripheral and peripheral perivascular leak. At age 17 years, (D) the MRI cervical spine, T2 demonstrated severe spinal canal stenosis with deformation of the spinal cord and cord signal abnormality, suggestive of compressive myelopathy. Enzyme replacement therapy (ERT) was started at age 18 years and by age 20 years, (E) there was complete resolution of the vitreous hemorrhage in the right eye and (F) there was reduced mid peripheral and peripheral perivascular leak in the left eye. Accordingly, (G) the biomarkers chitotriosidase (CHITO), angiotensin‐converting enzyme (ACE), Glucosylsphingosine (Lyso‐GB1), and tartrate resistant acid phosphatase (TRAP) decreased after enzyme replacement therapy initiation.

A 20‐year‐old American Hispanic female born to distantly related parents (fifth degree relatives) first presented at age 11 years to an ophthalmology clinic for myopic astigmatism and blepharitis. At age 14 years, the patient developed blurry vision and was found to have intermediate uveitis with active intraocular inflammation, vitreous hemorrhage and cystoid macular edema in the right eye, and bilateral epiretinal membranes (Figure 1B,C,E,F). Her best corrected visual acuity was 20/400 in the right eye and 20/20 in the left eye. A uveitis workup for systemic autoimmune diseases as well as infectious conditions was notable for a platelet count of 92 000/μL and a moderately high‐titer positive anti‐nuclear antibody at 1:640 with a centromere staining pattern. She had an elevated serum angiotensin‐converting enzyme (ACE) at 116 nmol/mL/min (reference range <40). Anti‐dsDNA, anti‐Smith, anti‐ribonucleoprotein, anti‐RO, anti‐LA, rheumatoid factor, anticardiolipin, anti‐Beta2 glycoprotein, lupus anticoagulant, platelet antibody, hepatitis B, tuberculosis quantiferon gold, HLA‐B27, and antineutrophilic cytoplasmic antibody were all negative.

The patient was referred to pediatric rheumatology for further autoimmune workup as her initial treatment with topical and oral steroids failed to fully control her active intraocular inflammation. Additional workup of antiphospholipid antibodies, cytomegalovirus, Epstein–Barr virus, toxoplasmosis, human immunodeficiency virus, and autoantibody testing in the cerebrospinal fluid were all negative. Her presumed diagnosis was idiopathic intermediate uveitis, and she was started on an immunosuppression regimen with oral methotrexate 25 mg daily and adalimumab 40 mg every 14 days. The patient underwent magnetic resonance imaging (MRI) of the orbits and brain to rule out a demyelinating process that could cause uveitis and it revealed multiple non‐specific T2 hyperintense white‐matter foci (Figure 1A). A computerized tomography (CT) head angiogram was negative for vasculitides.

At age 15 years, the patient began experiencing new‐onset migraine headaches and she was referred to pediatric neurology who felt her symptoms may be explained by migraine with superimposed tension type headache. She was switched from adalimumab to infliximab 11 mg/kg once monthly for easier administration. At this time, the patient also began experiencing bilateral knee pain and effusion, diffuse myalgias and joint pains, as well as chest pain and dyspnea. Given the onset of new symptoms, sarcoidosis was considered as a possible cause given her persistent ACE elevation despite the initiation of anti‐tumor necrosis factor therapy. Due to her new symptoms of dyspnea and myalgia, pulmonary function testing (PFT), a 6‐min walk test, and tests for myositis were ordered to assess for pulmonary disease; all were non‐diagnostic.

At follow‐up with rheumatology at age 15 years, her uveitis and migraine symptoms seemed to have improved. However, on physical examination, her jaw opening aperture was greatly reduced. MRI of the temporomandibular joint was ordered and returned negative for juvenile idiopathic arthritis.

During follow‐up with rheumatology at age 17 years, the patient was found to have new‐onset limited range of motion (ROM) of neck, hypermobility of hands, stiff gait with genu valgum and pes planus, and a positive Gower's sign. MRI of the cervical spine was ordered for gait instability and demonstrated an acentric herniated nucleus pulposus and severe C3‐C4 stenosis with deformation of the spinal cord and signal abnormality suggestive of compressive myelopathy (Figure 1D). She was referred to hematology for work‐up to explain her thrombocytopenia where tests for Fanconi anemia, telomere length, and congenital platelet disorders were all non‐diagnostic. Shortly thereafter, the patient had a mechanical fall at school and begun to use a cane. She additionally experienced frequent joint subluxation with daily activities. X‐ray of bilateral femurs demonstrated Erlenmeyer flask deformity.

At age 18 years the patient was evaluated by neurosurgery to assess her gait instability and cervical stenosis. She was noted to have normal proprioception and normal tactile sensation but was found to have an ataxic gait. At this time, she was evaluated by pediatric neurology for follow up of cervical stenosis and her physical exam was notable for hyperreflexia and multiple myoclonic jerks. Electromyography (EMG) showed ongoing chronic distal motor polyneuropathy without signs of a cervical or lumbar radiculopathy.

Whole exome sequencing (WES) was ordered and revealed homozygosity for the c.1342G>C (p.Asp448His) variant in *GBA*, consistent with a diagnosis of GD type 3c. Familial testing revealed heterozygosity for the variant in the mother; the father was not available for testing. No additional genetic variants were reported. Evaluation by medical genetics at age 18 years revealed dysarthria, conjugate gaze with intermittent lateral‐gaze strabismus and upgaze palsy, wide‐based gait, proximal lower extremity weakness, and hyperreflexia with clonus. GCase levels were deficient in blood at 4.0 pmol/punch/h (normal limits >4.7). Glucosylsphingosine (lyso‐Gb1), was markedly elevated at 365.3 nmol/L (≤1.9^12^). In addition, platelets were low at 78 000/μL (150–450 000) and hemoglobin was low at 11.4 g/dL (12.0–15.5). ACE was 107 IU/L (25–106), chitotriosidase (CHITO) was 650 nmoles/h/mL (4–120), and tartrate resistant acid phosphatase (TRAP) was 28.8 IU/L (3–10)—all consistent with the diagnosis of GD^13^, ^14^ (Figure 1G). Abdominal MRI revealed splenomegaly with a volume of 314 mL (100 mL).

Intravenous (IV) imiglucerase treatment at 60 units/kg every 2 weeks was initiated and she was referred to pediatric cardiology for evaluation of cardiovascular manifestations of GD type 3c. An electrocardiogram demonstrated normal sinus rhythm with nonspecific T‐wave abnormalities. An echocardiogram demonstrated normal valve mobility, mild mitral valve thickening with mild hypoplasia and trace regurgitation. A CT coronary angiogram showed mild peripheral calcifications on the aortic sinus of Valsalva without involvement of the leaflets, normal cardiac function, and no evidence of coronary artery disease (Supplementary Table 1). Serial ambulatory heart rhythm monitors identified no significant ectopy or arrhythmias and she was monitored off of cardiac medications given her reassuring cardiac evaluation.

After 2 months of imiglucerase treatment, she reported decreased muscle spasms and possible improvements in mood and energy. However, the patient reported new‐onset liquid dysphagia and she was evaluated by speech language pathology (SLP). At that time, she was noted to have a weak cough and possible reduced laryngeal elevation. Despite a grossly normal barium swallow study, she continued to experience difficulty swallowing and subsequent weight loss due to poor oral intake.

At age 19 years, the patient experienced multiple hospitalizations. She was hospitalized for community‐acquired pneumonia likely due to aspiration and was readmitted 6 months later for a subsegmental pulmonary embolism, likely due to prolonged immobility and/or GD‐related pulmonary vasculature involvement. The patient's dysphagia progressed to include solid foods and gastroenterology suspected abnormal esophageal motility secondary to GD; a follow‐up barium swallow study was performed and again returned normal. Due to concerns about the patient's continued weight loss, which dropped from 59 to 47 kg over a period of 3 months resulting in a body mass index ([BMI]) of 16, a gastrostomy tube (G‐tube) was placed, and nutritional support was provided. Additionally, the patient was started on a daily dose of 20 mg of omeprazole to manage gastric reflux.

Also at age 19 years, the patient developed new onset of sialorrhea, and she was started on glycopyrrolate 2 mg three times a day. The sialorrhea was unresponsive to glycopyrrolate and the patient was then seen by otolaryngology where she received Botox injections to the bilateral parotid glands. At this time, the patient also experienced higher levels of anxiety attributable to her current symptoms and was started on citalopram 20 mg and trazodone 50 mg daily. The patient also began experiencing frequent nocturnal awakenings; she was referred to pulmonology who suspected a sleep‐related breathing disorder secondary to a limited oral aperture and a crowded upper airway. She was subsequently started on a mechanical insufflation‐exsufflation device twice daily to help with mucus clearance.

At this time the patient demonstrated progressive distal and proximal muscle weakness and a repeat EMG was performed. In comparison to the prior EMG study at age 17 years, there was progression of the previously identified systemic motor polyneuropathy (Supplementary Tables 2 and 3). Folate and B12 levels were normal to mildly elevated, at >22 ng/mL and 893 pg/mL respectively (reference: >6.5 ng/mL and 123–730 pg/mL). Serum neurofilament light chain levels were elevated at 4.78 pg/mL (0–1.59), which in the absence of vitamin deficiencies, suggested ongoing axonal damage. A follow‐up brain MRI to monitor for neurologic progression demonstrated stable white matter lesions with worsening ventriculomegaly.

After 1 year of treatment with ERT, the patient's ACE has decreased from 107 to 47 IU/L, CHITO from 650 to 10 nmol/h/mL, TRAP from 28.8 to 10.6 IU/L, and LysoGB1 from 365.3 to 56.9 nmol/L. Her hemoglobin remained stable at 12 g/dL and her platelets steadily increased to 207 000/μL. All immunosuppressive medications previously prescribed for uveitis were discontinued due to symptom improvement. At her most recent visit at age 20 years, she was more conversational and healthier than in the past. On physical exam, eye findings remained unchanged and her sialorrhea improved with Botox injections to the salivary gland. She continued to receive bolus G‐tube feedings in addition to some oral feeds which increased her BMI from 16 to 18. Ophthalmic examination revealed that her intraocular inflammation was well controlled, and her visual acuity improved to 20/30 in the right eye and 20/25 in the left eye. She had complete resolution of the vitreous hemorrhage and had mild residual retinal perivascular leak on wide field fluorescein angiograms which is being monitored without additional treatment.

Since some of the patient's symptoms had not been previously reported in GD type 3c, a chromosomal microarray (CMA) was ordered at age 20 years to rule out other potential genetic contributions to her unusual presentation. The CMA did not reveal any clinically relevant deletions or duplications but did confirm the known consanguinity (detected regions of homozygosity >6.2% of genome). Of note, *GBA* was located in a run of homozygosity involving chromosome 1q. WES reanalysis was also ordered and did not demonstrate any new findings that could explain her unique manifestations of GD type 3c. No genetic variants in the regions of homozygosity identified through CMA were identified.

## 3 DISCUSSION

We present an initial atypical presentation of GD type 3c with anterior uveitis and polyneuropathy, absent cardiac calcifications, and a significant delayed diagnosis of over 7 years after first presentation of symptoms with more than 10 subspecialists involved in her care. The delay in identifying GD type 3c could be attributed to unprecedented symptoms at presentation (chronic uveitis and polyneuropathy) without the aortic and mitral valve calcification often observed in GD type 3c, yet also represents how patients can have a long diagnostic odyssey despite clues to the features of a particular disease.

In our literature review, we focused on the clinical progression of GD type 3c, specifically highlighting the variability in its presentation and the extent of cardiac involvement. A total of 49 patients with homozygous c.1342G>C (p.Asp448His) variants in *GBA* have been reported in the literature to date (Table 1). A review of the diagnosis and management of 13 Turkish patients with GD type 3c^10^ provided a comprehensive analysis of the common symptoms and the age of onset for various complications associated with GD type 3c. Patients in this review had a median age at diagnosis of 12.3 years (range 10 months to 23 years), and most demonstrated classical GD type 3c findings of cardiac calcifications (11/13 patients, median age‐of‐onset 15.3 years), corneal opacities (10/13 patients), oculomotor apraxia (12/13 patients, median age‐of‐onset 14.167 years), with several experiencing psychiatric disorders (3/13 with ADHD, 4/13 with OCD, and 3/13 had abnormal psychometric evaluations).^10^ Concurrent with this review, our patient's age of diagnosis at 18 fell within the diagnosis range, and she additionally also demonstrated oculomotor apraxia. However, her unique neurologic manifestations of ongoing polyneuropathy, unique ophthalmological manifestations of chronic intermediate uveitis, and relative lack of cardiovascular calcifications have not been previously reported in this review or in other prior literature.^26^, ^27^

**TABLE 1:** Summary of presenting symptoms and cardiovascular involvement reported in patients with neurologic manifestations of homozygous c.1342G>C (p. Asp448His, historically referred to as D104H) variants in *GBA.*

Reference	Total *n* reported	Age at diagnosis (years)	Ethnicity	Presenting features	Neurologic manifestation	Cardiovascular manifestations
Chabas et al.^15^	*n* = 3 (siblings)	17	Spanish	(*n* = 2) Recurrent epistaxis, dyspnea on exertion, hepatosplenomegaly	(*n* = 2) Left ophthalmoplegia, saccadic eye movements, hyporeflexia, left strabismus	(*n* = 3) Ascending aorta and aortic/mitral valve calcification
		16	Spanish			
		1.5^a^	Spanish	(*n* = 1) Hepatosplenomegaly	(n = 1) Myoclonic seizures	
Shah et al.^16^	*n* = 1	12	Indian	Fatigability, sinusitis, epistaxis, dyspnea on exertion, involuntary movements of upper limbs	Oculomotor apraxia	Calcification of endocardium, mitral and aortic valves, and aortic root and arch. Moderate mitral regurgitation, mild mitral stenosis, and mild aortic regurgitation
Abrahamov et al.^17^	*n* = 12	20	Saudi Arabian	(*n* = 1) Severe respiratory distress, splenomegaly^b^	(*n* = 1) Oculomotor apraxia	(*n* = 1) Severe aortic stenosis and insufficiency
Stone et al.^18^	*n* = 1	0.5	American	Splenomegaly, absent saccadic eye movements	Absent saccadic eye movements, communicating hydrocephalus, mild bilateral cerebral dysfunction	Ventricular hypertrophy, coronary artery and aortic intimal fibrosis
Bohlega et al.^19^	*n* = 4	16	Saudi Arabian	(*n* = 1) Abnormal eye movements associated with excessive blinking	(*n* = 1) Slow horizontal saccades, oculomotor apraxia, myoclonic jerks^b^	(*n* = 1) Mild cardiomegaly, ascending aorta, aortic root, and aortic and mitral valve calcification
Michelakakis et al.^20^	*n* = 1	0.63	Greek	Failure to thrive, hepatosplenomegaly	Generalized tonic–clonic seizures, hypertonia, oculomotor apraxia, hyperreflexia	Ventricular septal thickness
Cindik et al.^21^	*n* = 1	14	Turkish	Fatigue, palpitations, syncope	Oculomotor apraxia, hearing loss, tetra‐ventricular hydrocephalus	Massive atrial and left ventricular dilation, severe aortic and mitral valve insufficiency, aortic and mitral valve stenosis
Mireles et al.^11^	*n* = 1	13	Mexican	Chest pain and dyspnea on exertion	Hydrocephalus	Mild aortic stenosis and regurgitation, severe mitral stenosis and regurgitation, mild‐to‐moderate tricuspid regurgitation
Rastogi et al.^22^	*n* = 1	16	Indian	Easily fatigability, dyspnea, palpitations	Oculomotor apraxia	Hypertrophic left ventricle, an enlarged left atrium, mitral and aortic valve calcification with severe stenosis
Kurolap et al.^9^	*n* = 4	4	Israeli Muslim Arab	(*n* = 1) Oculomotor Apraxia, moderate intellectual disability	(*n* = 1) Oculomotor Apraxia, moderate intellectual disability	(*n* = 1) Aortic and other cardiac valve calcifications
		0	Israeli Muslim Arab	(*n* = 1) Opsoclonic eye movements, mild cognitive impairments	(*n* = 1) Opsoclonic eye movements, mild cognitive impairments	(*n* = 1) Severe ascending and descending aortic calcifications, pulmonary and mitral calcifications, severe pulmonic and mitral stenosis
		0.12	Spanish	(*n* = 1) Massive hepatosplenomegaly	(*n* = 1) Oculomotor apraxia	(*n* = 1) Severe aortic calcification and stenosis, coronary artery disease with diffuse lesions in the left main artery
Bulut et al.^10^	*n* = 13	0.83–23.75^c^	N/A	(*n* = 5) Neonatal cholestasis (*n* = 4) Other visceral involvement (*n* = 1) Neurologic involvement (*n* = 1) Cardiac involvement (*n* = 1) Other visceral involvement and hematological involvement	(*n* = 12) Oculomotor apraxia (*n* = 5) Cervical dystonia (*n* = 4) Epilepsy (*n* = 2) Myoclonus (*n* = 5) Fine tremor	Variable (11 of 13 with cardiac involvement); 1 demonstrated isolated aortic calcification, 2 with isolated cardiac valve calcifications, 5 with both cardiac valve and aortic calcifications, and 3 with left ventricular hypertrophy in addition to cardiac valve and aortic calcifications
Alsahli et al.^23^	*n* = 1	4	Saudi Arabian	Hepatosplenomegaly, pancytopenia	Oculomotor apraxia	Thickened mitral valve leaflets with severe mitral regurgitation, severely dilated left atrium, right ventricular outflow tract obstruction, diffuse thickening of the aortic root and aortic arch calcifications
Silva‐Estrada et al.^24^	*n* = 1	13	Mexican	Dyspnea on exertion, orthopnea	Oculomotor apraxia	Left ventricular hypertrophy, cardiomegaly, severe mitral and tricuspid regurgitation, ostial coronary, brachiocephalic, left carotid, left subclavian, aortic, aortic root, and mitral valve calcification
Pasmanik‐Chor et al.^25^	*n* = 12	5	Saudi Arabian	(*n* = 2) Oculomotor apraxia, systolic murmurs	(*n* = 3) Oculomotor apraxia	(*n* = 2) Moderate aortic stenosis
		6				
		20	Saudi Arabian	(*n* = 1) Oculomotor apraxia, splenomegaly, bicytopenia		(*n* = 1) Cardiac insufficiency, severe pulmonary hypertension, and aortic stenosis
Current study	*n* = 1	18	American Hispanic	Uveitis	Motor axonal neuropathy, oculomotor apraxia, ventriculomegaly, compressive myelopathy, white matter foci	Mild mitral valve thickening with mild hypoplasia and trace regurgitation. Mild peripheral calcifications on the aortic sinus of Valsalva without involvement of the leaflets, normal cardiac function

*Note*: A comprehensive literature search was conducted to investigate the characteristics of Gaucher disease type 3c with an emphasis on both cardiac and neurological manifestations. We searched PubMed and Embase databases for studies published through January 2024. MeSH terms included: “Gaucher” AND “neuronopathic” OR “Gaucher” AND “Disease” AND “3c” OR “Gaucher” AND “SUPRANUCLEAR GAZE PALSY” OR “Gaucher” AND “seizures” OR “Gaucher” AND “cardiovascular.” Through this search, we identified 99 papers. Then, we filtered these results to only include reports of those homozygous for c.1342G>C in *GBA* with relevant neurological features, and then we assessed phenotypic presentation of neurologic involvement as well as cardiac involvement. Through this narrowed search, we identified 15 relevant papers. We excluded articles that did not meet our criteria, including non‐English language publications, studies on patients without neurological features, and reports that dealt with only non‐neurological aspects of the disease. For patients described in pairs or in groups, we combined the information regarding their initial clinical presentation and cardiovascular manifestations to create a comprehensive overview of the disease's impact on the nervous system and cardiovascular health. All ages are provided in years.

Abbreviation: N/A, not available.

^a^ Proband of siblings.

^b^ Only index patient with individual information provided.

^c^ Only group data was provided, range is provided.

Here, we further characterize our patients' neurologic and ophthalmologic manifestations in the context of existing literature and provide possible pathophysiological mechanisms for their etiology. First, our patient's ophthalmologic findings, diagnosed as intermediate uveitis, was not found to be attributable to any other genetic or autoimmune cause, suggesting that her symptoms may be attributable to GD type 3c. Recent studies have highlighted the critical role of the inflammatory complex in the pathogenesis of Gaucher disease. The accumulation of glucocerebroside in macrophages triggers a cascade of inflammatory responses. These macrophages secrete pro‐inflammatory cytokines such as interleukin‐1beta, tumor necrosis factor‐alpha, and interleukin‐6, which contribute to a chronic inflammatory state. This inflammation can manifest in various ways, depending on the tissue affected^28^ and the severity of the enzyme deficiency.^29^, ^30^ Inflammatory mediators and cells may infiltrate the uveal tract (comprising the iris, ciliary body, and choroid), leading to uveitis. This ocular involvement can be part of the broader spectrum of clinical manifestations of Gaucher Disease, especially in the setting of significant systemic disease as was noted in our patient and also in previously reported in a patient with GD type 1.^26^

In the context of neurologic manifestations of GD type 3c, oculomotor apraxia and supranuclear gaze palsy, but not polyneuropathy, has been previously documented. In fact, a prior case series report by Andreasson et al.^31^ did not find a definitive relationship between polyneuropathy and any subtype of GD type 3. In their study, three adult patients who screened positive for polyneuropathy (PNP) symptoms received subsequent examinations with the Utah Early Neuropathy Scale (UENS) and underwent electrodiagnostic testing with sensorimotor nerve conduction studies. Only one of the 3 patients was identified to have a mild large fiber PNP, but they also suffered from concurrent rheumatoid arthritis and polymyalgia rheumatica, which may have confounded whether PNP was due to GD type 3. This is similar to our case where the initial consideration was an autoimmune process due to the presence of uveitis and other symptoms. The role of inflammation as a contributory factor to the pathomechanisms of GD could represent a fruitful target for further research.

Although we speculate her ocular and neurologic involvement were directly attributable to GD type 3c, we also acknowledge that these findings may be unrelated. Whole‐exome sequencing (WES) and microarray analysis did not identify any additional genetic or autoimmune factors, but we cannot rule out the possibility of another underlying genetic or multifactorial cause for these manifestations.

We further consider the possibility that these symptoms might require treatments different from those typically used for GD type 3c. Although ERT has been shown to improve the systemic manifestations of GD type 3c, it has not been shown to impact patients' neurological, ophthalmologic, or cardiac symptoms.^20^, ^32^ As such, our patient exhibited improvement in laboratory markers indicative of systemic involvement. Interestingly, our patient's uveitis has shown improvement while on ERT and with cessation of her prior immunomodulatory medication. Her improvement may be attributable to the reduction in proinflammatory cytokines associated with ERT.^33^

In summary, the documentation of more cases in the literature will be essential to enhancing our understanding of the full phenotypic spectrum of GD type 3c and its varied clinical presentations. The delayed diagnosis of GD type 3c in our patient underscores the significance of maintaining a high index of suspicion for broader diagnoses that may explain multiple systemic symptoms. Although our patient had an atypical presentation of GD type 3c, there were several clinical findings and lab values consistent with the spectrum of GD; a low hemoglobin, thrombocytopenia, an increase in serum ACE levels, Erlenmeyer flask deformity on X‐ray, and MRI white matter hyperintensity changes, highlighting the need for multidisciplinary evaluation and comprehensive genetic analysis. This includes investigating other potential genetic and non‐genetic causes of unreported or atypical symptoms, especially in the setting of known or potential consanguinity. In addition, one future consideration could be to immunophenotype GD patients to further characterize the role of inflammation as a secondary effector of storage diseases.^34^ Further reports on outcomes of patients treated with ERT are needed for a better understanding of its therapeutic efficacy in GD type 3c. Continued data sharing and case reports are necessary to broaden our understanding of the full spectrum of GD type 3c and to develop appropriate treatment and management plans for patients across their lifespans. Broadly, patients with non‐typified inflammatory processes alongside chronic evolution of their symptoms may benefit from a comprehensive genetic study to identify possible etiologies for their condition.

## AUTHOR CONTRIBUTIONS

*Supervision*: PSK. *Writing original draft, Literature review*: JSW. *Writing, review, and editing*: JSW, RLK, SSS, EH, APL, DSG, DKJ, and PSK.

## FUNDING INFORMATION

The authors declare no funding sources.

## CONFLICT OF INTEREST STATEMENT

John S. Wang, Rebecca L. Koch, Daniel Kenney‐Jung, Erin Huggins, Sirajbir S. Sodhi, Andrew P. Landstrom, Dilraj S. Grewal, and Priya S. Kishnani declare that the research was conducted in the absence of any commercial or financial relationships that could be construed as a potential conflict of interest. Priya S. Kishnani has received honoraria and grant support from Sanofi and Takeda Pharmaceuticals.

## ETHICS STATEMENT

Ethics approval was not required for this case report.

## INFORMED CONSENT STATEMENT

This case report was conducted in accordance with the Duke University Health System (DUHS) Institutional Review Board (IRB). The review of medical records for publication of a single case report is not considered by the DUHS IRB to be research involving human subjects, and therefore this medical case report did not require IRB review and approval. The collection and evaluation of all protected patient health information was performed in a Health Insurance Portability and Accountability Act (HIPAA)‐compliant manner. All procedures followed were in accordance with the ethical standards of the responsible committee on human experimentation (institutional and national) and with the Helsinki Declaration of 1975, as revised in 2000 (5). Informed consent was obtained from the patient for being included in the study.

## Supporting information


**Supplementary Table 1.** Summary of neuroimaging and functional testing results of our patient with Gaucher disease type 3c.
**Supplementary Table 2.** Abnormal electromyography findings at age 19 years (1 year after enzyme replacement therapy) demonstrate ongoing motor axonal polyneuropathy.
**Supplementary Table 3.** Motor nerve conduction study at age 19 years (1 year after initiating enzyme replacement therapy).

## Data Availability

The original contributions presented in the case report are included in the article. Further inquiries can be directed to the corresponding author.

## References

[1] Dionisi‐Vici C , Rizzo C , Burlina AB , et al. Inborn errors of metabolism in the Italian pediatric population: a national retrospective survey. J Pediatr. 2002;140(3):321‐327. 11953730 10.1067/mpd.2002.122394

[2] Kilavuz S , Basaranoglu M , Epcacan S , et al. A rare cause of hydrops fetalis in two Gaucher disease type 2 patients with a novel mutation. Metab Brain Dis. 2022;37(4):1283‐1287. 35254599 10.1007/s11011-022-00942-5

[3] Weiss K , Gonzalez AN , Lopez G , Pedoeim L , Groden C , Sidransky E . The clinical management of type 2 Gaucher disease. Mol Genet Metab. 2015;114(2):110‐122. 25435509 10.1016/j.ymgme.2014.11.008 PMC4312716

[4] Stirnemann J , Belmatoug N , Camou F , et al. A review of Gaucher disease pathophysiology, clinical presentation and treatments. Int J Mol Sci. 2017;18(2):10‐11. 10.3390/ijms18020441 PMC5343975 28218669

[5] Schwartz IVD , Göker‐Alpan Ö , Kishnani PS , et al. Characteristics of 26 patients with type 3 Gaucher disease: a descriptive analysis from the Gaucher outcome survey. Mol Genet Metab Rep. 2018;14:73‐79. 29326879 10.1016/j.ymgmr.2017.10.011 PMC5758841

[6] Sestito S , Filocamo M , Ceravolo F , et al. Norrbottnian clinical variant of Gaucher disease in southern Italy. J Hum Genet. 2017;62(4):507‐511. 28003644 10.1038/jhg.2016.158

[7] Brady RO , Barton NW , Grabowski GA . The role of neurogenetics in Gaucher disease. Arch Neurol. 1993;50(11):1212‐1224. 8215980 10.1001/archneur.1993.00540110088009

[8] Patterson MC , Horowitz M , Abel RB , et al. Isolated horizontal supranuclear gaze palsy as a marker of severe systemic involvement in Gaucher's disease. Neurology. 1993;43(10):1993‐1997. 8413956 10.1212/wnl.43.10.1993

[9] Kurolap A , del Toro M , Spiegel R , et al. Gaucher disease type 3c: new patients with unique presentations and review of the literature. Mol Genet Metab. 2019;127(2):138‐146. 31130326 10.1016/j.ymgme.2019.05.011

[10] Bulut FD , Kor D , Kılavuz S , et al. Expanding the phenotypic landscape of Gaucher disease type 3c with a novel entity ‐ transient neonatal cholestasis. Eur J Med Genet. 2023;66(6):104764. 37061027 10.1016/j.ejmg.2023.104764

[11] Mireles SA , Seybold J , Williams G . Undiagnosed type IIIc Gaucher disease in a child with aortic and mitral valve calcification: perioperative complications after cardiac surgery. J Cardiothorac Vasc Anesth. 2010;24(3):471‐474. 19632857 10.1053/j.jvca.2009.05.006

[12] Revel‐Vilk S , Fuller M , Zimran A . Value of glucosylsphingosine (Lyso‐Gb1) as a biomarker in Gaucher disease: a systematic literature review. Int J Mol Sci. 2020;21(19):12. 10.3390/ijms21197159 PMC7584006 32998334

[13] Giuffrida G , Markovic U , Condorelli A , et al. Glucosylsphingosine (Lyso‐Gb1) as a reliable biomarker in Gaucher disease: a narrative review. Orphanet J Rare Dis. 2023;18(1):27. 36782327 10.1186/s13023-023-02623-7 PMC9926807

[14] Stiles AR , Huggins E , Fierro L , Jung SH , Balwani M , Kishnani PS . The role of glucosylsphingosine as an early indicator of disease progression in early symptomatic type 1 Gaucher disease. Mol Genet Metab Rep. 2021;27:100729. 33614410 10.1016/j.ymgmr.2021.100729 PMC7876627

[15] Chabas A , Cormand B , Grinberg D , et al. Unusual expression of Gaucher's disease: cardiovascular calcifications in three sibs homozygous for the D409H mutation. J Med Genet. 1995;32(9):740‐742. 8544197 10.1136/jmg.32.9.740 PMC1051678

[16] Shah S , Misri A , Bhat M , Maheshwari S . Gaucher's disease type III C: unusual cause of intracardiac calcification. Ann Pediatr Cardiol. 2008;1(2):144‐146. 20300259 10.4103/0974-2069.43883 PMC2840747

[17] Abrahamov A , Elstein D , Zimran A , et al. Gaucher's disease variant characterised by progressive calcification of heart valves and unique genotype. Lancet. 1995;346(8981):1000‐1003. 7475546 10.1016/s0140-6736(95)91688-1

[18] Stone DL , Tayebi N , Coble C , Ginns EI , Sidransky E . Cardiovascular fibrosis, hydrocephalus, ophthalmoplegia, and visceral involvement in an American child with Gaucher disease. J Med Genet. 2000;37(11):E40‐E440. 11073549 10.1136/jmg.37.11.e40 PMC1734463

[19] Bohlega S , Kambouris M , Shahid M , al Homsi M , al Sous W . Gaucher disease with oculomotor apraxia and cardiovascular calcification (Gaucher type IIIC). Neurology. 2000;54(1):261‐263. 10636167 10.1212/wnl.54.1.261

[20] Michelakakis H , Skardoutsou A , Mathioudakis J , et al. Early‐onset severe neurological involvement and D409H homozygosity in Gaucher disease: outcome of enzyme replacement therapy. Blood Cells Mol Dis. 2002;28(1):1‐4. 11814305 10.1006/bcmd.2001.0477

[21] Cindik N , Ozcay F , Süren D , et al. Gaucher disease with communicating hydrocephalus and cardiac involvement. Clin Cardiol. 2010;33(1):E26‐E30. 19816973 10.1002/clc.20348 PMC6653081

[22] Rastogi P , Rao S , Kaur J , Malhotra P , Varma S , das R . Gaucher's disease with cardiac valve calcification and stenosis: a rare presentation due to homozygous p.D409H mutation in a north Indian family. Indian J Pediatr. 2016;83(8):877‐878. 26887759 10.1007/s12098-015-2025-7

[23] Alsahli S , Bubshait DK , Rahbeeni ZA , Alfadhel M . Aortic calcification in Gaucher disease: a case report. Appl Clin Genet. 2018;11:107‐110. 30410382 10.2147/TACG.S180995 PMC6199969

[24] Silva‐Estrada J , Cervantes‐Barragán DE , Reyes‐de la Cruz L , Meléndez‐Ramírez G , Meave A , Alaez‐Verson C . Pediatric porcelain aorta secondary to Gaucher disease type 3C with successful aortic replacement surgery. JACC Case Rep. 2022;4(22):1504‐1508. 36444188 10.1016/j.jaccas.2022.08.020 PMC9700068

[25] Pasmanik‐Chor M , Laadan S , Elroy‐Stein O , et al. The glucocerebrosidase D409H mutation in Gaucher disease. Biochem Mol Med. 1996;59(2):125‐133. 8986634 10.1006/bmme.1996.0077

[26] Dweck A , Rozenman J , Ronen S , Zimran A , Elstein D . Uveitis in Gaucher disease. Am J Ophthalmol. 2005;140(1):146‐147. 16038664 10.1016/j.ajo.2004.12.081

[27] Raz J , Anteby I , Livni N , Benezra D . Chronic uveitis in Gaucher's disease. Ocul Immunol Inflamm. 1993;1(1–2):119‐124. 22827202 10.3109/09273949309086547

[28] Barak V , Acker M , Nisman B , et al. Cytokines in Gaucher's disease. Eur Cytokine Netw. 1999;10(2):205‐210. 10400826

[29] Basiri M , Ghaffari ME , Ruan J , et al. Osteonecrosis in Gaucher disease in the era of multiple therapies: biomarker set for risk stratification from a tertiary referral center. Elife. 2023;12:12. 10.7554/eLife.87537 PMC10317498 37249220

[30] Sahasrabudhe SA , Terluk MR , Rudser KD , Cloyd JC , Kartha RV . Biological variation in peripheral inflammation and oxidative stress biomarkers in individuals with Gaucher disease. Int J Mol Sci. 2022;23(16):8‐9. 10.3390/ijms23169189 PMC9409136 36012454

[31] Andreasson M , Solders G , Björkvall CK , Machaczka M , Svenningsson P . Polyneuropathy in Gaucher disease type 1 and 3—a descriptive case series. Sci Rep. 2019;9(1):15358. 31653957 10.1038/s41598-019-51976-2 PMC6814858

[32] van Dussen L , Biegstraaten M , Dijkgraaf MGW , Hollak CEM . Modelling Gaucher disease progression: long‐term enzyme replacement therapy reduces the incidence of splenectomy and bone complications. Orphanet J Rare Dis. 2014;9:112. 25056340 10.1186/s13023-014-0112-x PMC4226965

[33] Matta MC , Vairo F , Torres LC , Schwartz I . Could enzyme replacement therapy promote immune tolerance in Gaucher disease type 1? Blood Cells Mol Dis. 2018;68:200‐202. 28029576 10.1016/j.bcmd.2016.10.016

[34] Pandey MK . Exploring pro‐inflammatory immunological mediators: unraveling the mechanisms of neuroinflammation in lysosomal storage diseases. Biomedicine. 2023;11(4):200‐201. 10.3390/biomedicines11041067 PMC10135900 37189685

