# Supplementary material for: Gaucher disease type 3c: Expanding the clinical spectrum of an ultra‐rare disease
Source: JIMD Rep. 2024 Aug 15;65(5):313–22. doi: 10.1002/jmd2.12440 (PMC11558466; doi:10.1002/jmd2.12440)
Supplement: Supplementary file 1 — Supplementary Table 1. Summary of neuroimaging and functional testing results of our patient with Gaucher disease type 3c. Supplementary Table 2. Abnormal electromyography findings at age 19 years (1 year after enzyme replacement therapy) demonstrate ongoing motor axonal polyneuropathy. Supplementary Table 3. Motor nerve conduction study at age 19 years (1 year after initiating enzyme replacement therapy). [file JMD2-65-313-s001.docx]

# Supplementary Table 1: Summary of neuroimaging and functional testing results of our patient with Gaucher disease type 3c.

| **Age (years)** | **Procedure Type** | **Summary of Findings** |
| --- | --- | --- |
| 14 | MRI of the orbits | Multiple non-specific T2 hyperintense white-matter foci. |
| 14 | MRI brain without Contrast | Multiple nonspecific small foci of white matter T2/FLAIR hyperintensity within right greater than left cerebral hemispheres. Presence of thin membrane-like T2 signal abnormality within the posterior chamber of the right orbit. Bilateral optic nerves unremarkable. |
| 15 | CT head angiogram | No evidence of vasculitis of the large and medium size intracranial vessels. |
| 15 | MRI temporomandibular joints | No evidence of TMJ arthritis |
| 17 | MRI cervical spine | C3-C4 posterior right eccentric disc extrusion with posterior osteophytes, resulting in severe spinal canal stenosis with deformation of the spinal cord and cord signal abnormality, suggestive of compressive myelopathy. |
| 18 | ERT initiated | |
| 18 | X-ray fluoroscopy video swallow with speech | No penetration or aspiration (Limited trials per patient's preference). |
| 18 | Electromyography | Ongoing denervation and chronic reinnervation changes were seen in the left tibialis anterior. Chronic reinnervation changes were seen in the left first dorsal interossei (FDI). |
| 19 | Echocardiography | Normal left and right ventricular systolic function, normal pressures with normal diastolic function. Trivial to mild regurgitation and no valvular stenosis. |
| 19 | X-ray barium swallow | Mild esophageal dysmotility seen on upright views. Esophageal motility not assessed as patient was unable to drink liquids while in prone positioning. |
| 19 | Electromyography | Active denervation changes as well as chronic reinnervation changes in the right abductor pollicis brevis (APB) and bilateral FDI muscles with abundant spontaneous activity in addition to motor unit potential abnormalities. Right upper extremity showed chronic reinnervation changes in the right triceps, extensor digitorum, and pronator teres muscles with tall, wide motor unit potentials as well as reduced recruitment and polyphasia. Left upper extremity showed chronic reinnervation changes in the left triceps, and extensor digitorum muscles with tall, wide motor unit potentials, reduced recruitment, and polyphasia. |
| 19 | MRI Brain without contrast | Unchanged number of numerous non-enhancing supratentorial white matter lesions. Worsening of the patient's known ventriculomegaly which may represent hydrocephalus. |

| **Side** | **Muscle** | **Fibrillations** | **Positive Sharp Waves** | **Amplitude** | **Duration** | **Polyphasic Potentials** | **Recruitment** |
| --- | --- | --- | --- | --- | --- | --- | --- |
| **Right** | Triceps | None | None | 2+ | 2+ | Normal | 1- |
|  | First Dorsal Interossei (FDI) | 3+ | 3+ | 2+ | 2+ | Normal | 2- |
|  | Extensor Digitorum (ED) | None | None | 1+ | 1+ | Many | 1- |
|  | Pronator Teres (PT) | None | None | 1+ | Normal | Normal | 1- |
|  | Abductor Pollicis Brevis (APB) | 3+ | 3+ | 1- | 2+ | Many | 3- |
| **Left** | Triceps | None | None | 2+ | 2+ | Normal | 2- |
|  | FDI | 3+ | 3+ | 2+ | 2+ | Normal | 2- |
|  | ED | None | None | 1+ | Normal | Few | Normal |

**Supplementary Table 2: Abnormal electromyography findings at age 19 years (one year after enzyme replacement therapy) demonstrate ongoing motor axonal polyneuropathy.**

Right and left upper extremity showed chronic reinnervation changes in the bilateral triceps, bilateral ED, right PT muscles with tall, wide motor unit potentials, reduced recruitment, and polyphasia. There were active denervation changes and chronic reinnervation changes in the right APB and bilateral FDI with abundant spontaneous activity in addition to motor unit potential abnormalities. NA = not available.

# Supplementary Table 3: Motor nerve conduction study at age 19 years (one year after initiating enzyme replacement therapy).

| **Site** | **Latency** | | **Amplitude** | | **Duration** | **F-Latency** | **Conduction Velocity** | |
| --- | --- | --- | --- | --- | --- | --- | --- | --- |
|  | **(ms)** | **Reference** | **(mV)** | **Reference** | **(ms)** | **(ms)** | **(m/s)** | **Reference** |
| **Left Median (APB) Motor** | | | | | | | | |
| Wrist | 4.7 | <4.4 | 2.4 | >4.2 | 6.5 | 31.9 | NA | NA |
| Elbow | 9.5 | NA | 2.5 | >4.2 | 6.6 | NA | 44 | >49 |
| **Right Median (APB) Motor** | | | | | | | | |
| Wrist | 4.8 | <4.4 | 1.51 | >4.2 | 5 | No Response | NA | NA |
| Elbow | 8.7 | NA | 1.37 | >4.2 | 6.3 | NA | 51 | >49 |
| **Left Ulnar (ADM) Motor** | | | | | | | | |
| Wrist | 3.2 | <3.5 | 7.1 | >5.6 | 4.6 | 29.6 | NA | NA |
| Below Elbow | 6.6 | NA | 6.5 | >5.6 | 4.9 | NA | 56 | >49 |
| Above Elbow | 9.1 | NA | 4.7 | >5.6 | 5 | NA | 49 | NA |
| **Right Ulnar (ADM) Motor** | | | | | | | | |
| Wrist | 3.3 | <3.5 | 5.8 | >5.6 | 4.6 | 29.9 | NA | NA |
| Below Elbow | 6.8 | NA | 4.9 | >5.6 | 4.9 | NA | 57 | >49 |
| Above Elbow | 8.7 | NA | 4.2 | >5.6 | 5.7 | NA | 56 | NA |

Bilateral median motor responses to APB were abnormal. Left ulnar motor response to abductor digiti minimi (ADM) displayed low amplitude above the elbow. Right ulnar motor response to ADM showed low amplitude across both elbow sites. NA = not available.
